# Efficacy and safety of proton pump inhibitors for stress ulcer prophylaxis in critically ill patients: a systematic review and meta-analysis of randomized trials

**DOI:** 10.1186/s13054-016-1305-6

**Published:** 2016-05-04

**Authors:** Fayez Alshamsi, Emilie Belley-Cote, Deborah Cook, Saleh A. Almenawer, Zuhoor Alqahtani, Dan Perri, Lehana Thabane, Awad Al-Omari, Kim Lewis, Gordon Guyatt, Waleed Alhazzani

**Affiliations:** Department of Medicine, McMaster University, Hamilton, Canada; Department of Internal Medicine, United Arab Emirates University, Alain, United Arab Emirates; Department of Clinical Epidemiology & Biostatistics, McMaster University, Hamilton, Canada; Department of Surgery, Division of Neurosurgery, McMaster University, Hamilton, Canada; Department of Critical Care, Security Forces Hospital, Riyadh, Saudi Arabia; Department of Medicine, Alfaisal University, Riyadh, Saudi Arabia; Department of Medicine, Division of Critical Care, St Joseph’s Healthcare, 50 Charlton Avenue East, Hamilton, ON L8N 4A6 Canada

## Abstract

**Background:**

The relative efficacy and safety of proton pump inhibitors (PPIs) compared to histamine-2-receptor antagonists (H2RAs) should guide their use in reducing bleeding risk in the critically ill.

**Methods:**

We searched the Cochrane library, MEDLINE, EMBASE, ACPJC, clinical trials registries, and conference proceedings through November 2015 without language or publication date restrictions. Only randomized controlled trials (RCTs) of PPIs vs H2RAs for stress ulcer prophylaxis in critically ill adults for clinically important bleeding, overt gastrointestinal (GI) bleeding, nosocomial pneumonia, mortality, ICU length of stay and *Clostridium difficile* infection were included. We used the Grading of Recommendations Assessment, Development and Evaluation (GRADE) approach to assess our confidence in the evidence for each outcome.

**Results:**

In 19 trials enrolling 2117 patients, PPIs were more effective than H2RAs in reducing the risk of clinically important GI bleeding (RR 0.39; 95 % CI 0.21, 0.71; *P* = 0.002; *I*^*2*^ = 0 %, moderate confidence) and overt GI bleeding (RR 0.48; 95 % CI 0.34, 0.66; *P* < 0.0001; *I*^*2*^ = 3 %, moderate confidence). PPI use did not significantly affect risk of pneumonia (RR 1.12; 95 % CI 0.86, 1.46; *P* = 0.39; *I*^*2*^ = 2 %, low confidence), mortality (RR 1.05; 95 % CI 0.87, 1.27; *P* = 0.61; *I*^*2*^ = 0 %, moderate confidence), or ICU length of stay (mean difference (MD), –0.38 days; 95 % CI –1.49, 0.74; *P* = 0.51; *I*^*2*^ = 30 %, low confidence). No RCT reported Clostridium difficile infection.

**Conclusions:**

PPIs were superior to H2RAs in preventing clinically important and overt GI bleeding, without significantly increasing the risk of pneumonia or mortality. Their impact on Clostridium difficile infection is yet to be determined.

**Electronic supplementary material:**

The online version of this article (doi:10.1186/s13054-016-1305-6) contains supplementary material, which is available to authorized users.

## Background

Over four decades ago, investigators first described stress ulcer bleeding in critically ill patients [1]. Since then, multiple studies have described this condition and its impact on the prognosis of critically ill patients. Stress ulcers typically occur in the gastric body, esophagus, or duodenum, sometimes resulting in gastrointestinal (GI) bleeding. Earlier studies reported overt GI bleeding in 5 to 25 % of critically ill patients [2, 3]. In contrast, the incidence of clinically important GI bleeding is much lower, estimated between 1 and 4 % [2, 4–7]. A recent large observational study (1034 patients, 97 sites), reported a 2.6 % incidence of clinically important GI bleeding [7], which was previously found to be associated with increased intensive care unit (ICU) mortality and length of stay [8]. Despite reduction in clinically important GI bleeding, pharmacologic stress ulcer prophylaxis does not seem to affect mortality in randomized controlled trials (RCTs) [8].

RCTs have investigated different classes of medication for stress ulcer prophylaxis. Recently, a meta-analysis of 29 RCTs showed that prophylaxis with either proton pump inhibitors (PPIs) or histamine-2-receptor antagonists (H2RAs) was associated with lower risk of overt GI bleeding compared to placebo or no prophylaxis [9]. However, the relative effectiveness of the two classes of agent remains uncertain.

PPIs, more potent at increasing gastric pH than H2RAs and maintaining gastric pH between 3.5 and 5.0, may minimize the risk of gastric mucosal injury [10]. Of four meta-analyses comparing PPIs to H2RAs, three suggested that PPIs are superior to H2RAs [11–13] and one did not [14].

The Surviving Sepsis Campaign (SSC) guidelines recommend using stress ulcer prophylaxis among critically ill patients with risk factors (e.g., mechanically ventilated patients, and patients with coagulopathy), including a weak recommendation for using PPIs over H2RAs in this setting [15]. The advice is concordant with current practice: recent observational studies showed that PPIs are the most commonly used prophylactic agents in the ICU [16–18].

In terms of the relative impact of PPIs and H2RAs, adverse effects are also a concern. In particular, a recent large retrospective observational study suggested PPI versus H2RA use in critically ill patients was associated with higher risks of pneumonia and *Clostridium difficile* infection compared to H2RA [19]. These results are, however, limited by the observational study design.

Several RCTs have been published recently and may influence both risk of bias and precision [20–25]. Therefore, we conducted a systematic review and meta-analysis to evaluate the efficacy and safety of PPIs compared to H2RAs for stress ulcer prophylaxis in critically ill patients. We used the Grading of Recommendations Assessment, Development and Evaluation (GRADE) methodology to assess the quality of evidence [26].

## Methods

### Study selection

Studies were eligible if: (1) the study design was an RCT; (2) the population involved adult critically ill patients in the ICU; (3) the intervention group received a PPI (either parenteral or enteral), regardless of the dose, frequency, or duration; (4) the control group received an H2RA, either parenteral or enteral, regardless of the dose, frequency, or duration; and (5) the outcomes included all or any of the following: clinically important GI bleeding; overt upper GI bleeding; pneumonia; mortality, ICU length of stay, and/or *Clostridium difficile* infection.

### Search strategy

We updated our previous systematic review [12] and searched MEDLINE, EMBASE, Cochrane Library, ACPJC, and International Clinical Trial Registry Platform (ICTRP) from March 2012 through November 2015. Our search strategy is detailed in Additional file 1: Tables S3-S5. We screened citations of all new potentially eligible articles without language or publication date restrictions. We conducted an electronic search of conference proceedings via a website provided by McMaster University (http://library.mcmaster.ca/articles/proceedingsfirst). Two reviewers (FA and EB) screened titles and abstracts to identify articles for full review, and evaluated the full text of potentially eligible studies. Disagreements between reviewers were resolved by consensus, and if necessary, consultation with a third reviewer (WA).

### Data extraction

Two reviewers (FA and EB) independently extracted pertinent data from all new studies utilizing a pre-designed data abstraction form. Disagreements were resolved by discussion and consensus. We contacted study authors for missing or unclear information.

### Risk of bias assessment

Two reviewers (FA and EB) independently examined eligible trials for risk of bias using the Cochrane Collaboration tool [27]. For each included trial, we judged articles as having low, unclear, or high risk of bias for the domains of adequate sequence generation, allocation sequence concealment, blinding for objective outcomes, incomplete outcome data, selective outcome reporting, and for other bias. The overall risk of bias for each trial included was categorized as low if the risk of bias was low in all domains, unclear if the risk of bias was unclear in at least one domain and with no high risk of bias domain, or high if the risk of bias was high in at least one domain. We resolved disagreements by discussion and consensus.

### Statistical analysis

We analyzed data using RevMan software (Review Manager, version 5.3. Copenhagen: The Nordic Cochrane Centre, The Cochrane Collaboration, 2014). We used the DerSimonian and Laird [28] random-effects model to pool the weighted effect of estimates across all studies. We estimated study weights using the inverse variance method. We calculated pooled relative risks (RRs) for dichotomous outcomes and mean differences (MDs) for continuous outcomes, with corresponding 95 % confidence intervals (CIs). We assessed statistical heterogeneity using Chi^2^ and *I*^2^ statistics [29]. We predefined substantial heterogeneity as *P* < 0.10 *or I*^*2*^ > 50 %.

We calculated the number needed to treat (NNT) using the method proposed by the Cochrane Collaboration [30]. We used an assumed control group (ACR) event rate of 3 % for clinically important bleeding and 5 % for overt GI bleeding; these ACRs were based on the results of a recent observational study [7]. We inspected funnel plots and performed Egger’s test to assess publication bias [31]. We explored heterogeneity between studies by performing predetermined subgroup analyses to investigate whether certain factors influenced the treatment effect. These subgroups included high vs. low risk of bias (hypothesizing that studies with high risk of bias would have a larger treatment effect), PPI route of administration (hypothesizing that the treatment effect would be larger with parenteral administration), PPI dose (hypothesizing that treatment effect would be larger with higher dosing). In addition, we conducted a post hoc sensitivity analysis, excluding trials published in abstract form [20, 23, 32–35].

## Results

### Characteristics of studies included

**Fig. 1:**
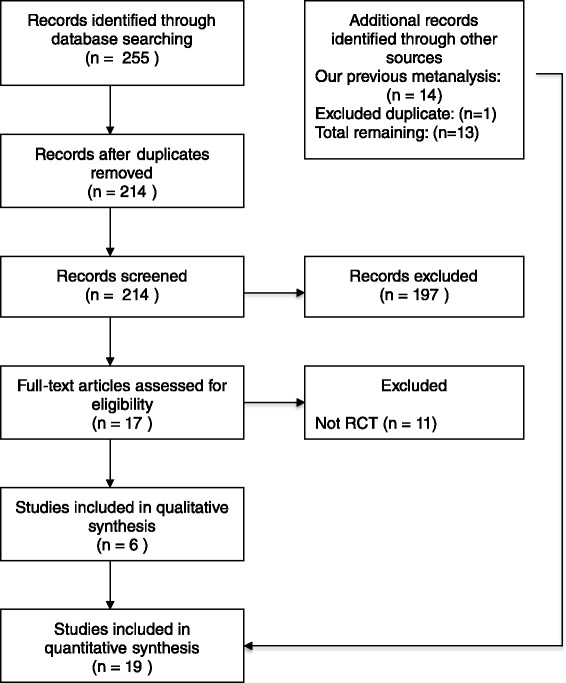
Process of identifying eligible studies: 18 trials (5 abstracts and 13 full published articles) were eligible and were included in the qualitative and quantitative analyses. *RCT* randomized controlled trial

Our new search identified a total of 255 citations. After removing duplicates, 214 articles remained. Following screening of titles and abstracts, 197 articles were excluded; 17 articles were retrieved for full text assessment and 11 were excluded for variable reasons (Fig. 1). After reviewing our previous results, we excluded an abstract [36] (*n* = 202) that was later published as a full article [35]. Another study was published as an abstract but was excluded from the analysis because the necessary data could not be obtained [37]. A total of six new trials (*n* = 600 patients) are included in the different analyses.

Combining our previous and current results, 19 RCTs [20, 22–25, 32–35, 38–48] from 20 reports (one study published outcomes separately in two different reports) [47, 48] met eligibility criteria and were included. Two eligible trials were published in abstract form [32, 33]; further information was obtained after contacting the authors.

Of 19 eligible trials [20, 22–25, 32–35, 38–48], 6 were published as an abstract only [20, 23, 32–34, 38] (Table 1). Overall, the included RCTs enrolled 2117 critically ill patients with a wide spectrum of medical and surgical conditions. Ten trials used intravenous PPIs, and eight used enteral PPIs, and the route was not described in one trial, which was published in abstract form. [23] The definitions for bleeding and pneumonia varied across trials and are summarized in Table 1.

**Table 1:** Characteristics of trials included

Author	Population	Interventions	Definition of GI bleeding	Definition of pneumonia	Funding
Conrad [35] USA (*n* = 359)	MV patients with risk factors Age (mean) 55.6 years Male 59 % APACHE II (mean) 23.7	Omeprazole 40 mg IV twice daily loading, then 40 mg daily (*n* = 178) Cimetidine 300 mg IV bolus, then infusion at 50 mg/h (*n* = 181)	(1) Bright red blood not clearing after tube adjustment and lavage (2) 8 h of persistent coffee grounds material with aspirates every 2 h not clearing with lavage or (3) Persistent coffee grounds material over 2–4 h on day 3–14 in 3 consecutive aspirates not clearing with lavage	USFDA	Pharmaceutical
Azevedo [36] Brazil (*n* = 108)	Critically ill patients with risk factors Age (mean) 56.7 years Male 52 % APAHE (mean) 55.3	Omeprazole 40 mg IV twice daily (*n* = 38) Ranitidine 150 mg/day IV (*n* = 38) Sucralfate 1 g PO four times daily (*n* = 32)	Overt bleeding	CDC criteria	NR
Hata [37] Japan (*n* = 210)	Cardiac surgery patients Age (mean) 64.5 years Male 73 % APACHE II NR	Rabeprazole 10 mg PO daily (*n* = 70) Ranitidine 300 mg PO daily (*n* = 70) Teprenone 150 mg NG daily (*n* = 70)	Overt bleeding with endoscopic lesions	NA	NR
Kantorova [38] Czech Republic (*n* = 287)	Surgical ICU with risk factors Age (mean) 47 years Male 67 % APACHE II (mean) 18.4	Omeprazole 40 mg IV daily (*n* = 72) Famotidine 40 mg IV twice daily (*n* = 71) Sucralfate 1 g PO four times daily (*n* = 69) Placebo (*n* = 75)	Overt bleeding with one of the following: (1) Drop in SBP >20 mmHg or rise in HR >20 beats/min within 24 h not explained by other causes or (2) Drop in hemoglobin >2 g/dL not explained by other causes	New or progressive infiltrate and 3 of the following: (1) Purulent ETT aspirate with >25 WBC/LPF (2) Peripheral leukocytosis >11 × 10^9^/or >10 % bands (3) Temperature >38.5 °C (4) Pathogen from aspirate, BAL (≥10^4^ CFU/mL) or protected brush sampling (≥10^3^ CFU/mL) (5) Positive blood or pleural cultures	Pharmaceutical
Kotlyanskaya [31] Abstract USA (*n* = 66)	MV patients. Age 71.2 years Male NR APACHE II 27.6	Lansoprazole (suspension) NG (*n* = 22) Lansoprazole (tablet) NG (*n* = 23) Ranitidine (*n* = 21) (dose and frequency not reported)	Overt bleeding associated with hemodynamic changes or Hb drop	NR	NR
Levy [39] USA (*n* = 67)	Medical and surgical ICU patients with risk factors. Age 57.1 years Male 55 % APACHE II 18.9	Omeprazole 40 mg NG daily (*n* = 32) Ranitidine 50 mg IV bolus, then 150 mg IV daily (*n* = 35)	Overt bleeding with hemodynamic instability, or a decrease Hb >2 g/dL requiring transfusion or associated with hemodynamic instability	NR	NR
Pan [40] China (*n* = 30)	Severe pancreatitis Age 48 years Male 45 % APACHE II 12.2	Rabeprazole 20 mg PO daily (*n* = 20) Famotidine 40 mg IV twice daily (*n* = 10)	Overt bleeding	NA	NR
Phillips [32] Abstract USA (*n* = 58)	MV patients with risk factors Age NR Male NR APACHE II NR	Omeprazole 40 mg PO, then 20 mg PO daily (*n* = 33) Ranitidine 50 mg IV loading, then 150–200 mg/day infusion (*n* = 25)	No clear definition	NR	NR
Powell [41] UK (*n* = 41)	Cardiac surgery Age 56.5 years Male 86 % APACHE II NR	Omeprazole 80 mg IV bolus, then 40 mg IV bolus three times daily (*n* = 10) Omeprazole 80 mg IV bolus then 40 mg IV infusion three times daily (*n* = 10) Ranitidine 50 mg IV three times daily (*n* = 11) Placebo (*n* = 10)	Overt bleeding	NA	Academic
Risaliti [42] Italy (*n* = 28)	Surgical ICU Age 61.5 years Male 64 % APACHE II NR	Omeprazole 40 mg IV daily, then 20 mg PO daily (*n* = 14) Ranitidine 150 mg IV daily, then 300 mg PO daily (*n* = 14)	No clear definition	NA	NR
Solouki [43] Iran (*n* = 129)	MV patients with other risk factors. Age 50.8 years Male 52 % APACHE II NR	Omeprazole 20 mg PO twice daily (*n* = 61) Ranitidine 50 mg IV twice daily (*n* = 68)	Overt bleeding associated with one of the following: (1) 20 mmHg decrease in SBP or DBP within 24 h or 20 beat/min increase in HR or postural drop by 10 mmHg in SBP (2) 2 g/dL decrease in Hb or 6 % decrease in Hct within 24 h (3) Lack of increase in Hb after two units of packed cells	New infiltrate and two of the following: (1) Fever ≥38.3 °C (2) WBC >10 × 10^9^/L (3) Pus in ETT aspirate	NR
Somberg [34] USA (*n* = 202)	Medical and surgical ICU patients with risk factors Age 42 years Male 74 % APACHE II 15.2	Pantoprazole 40 mg IV daily (*n* = 32) Pantoprazole 40 mg IV twice daily (*n* = 38) Pantoprazole 80 mg IV daily (*n* = 23) Pantoprazole 80 mg IV twice daily (*n* = 39) Pantoprazole 80 mg IV three times daily (*n* = 35) Cimetidine 300 mg IV bolus, then 50 mg/h infusion (*n* = 35)	(1) Hematemesis or bright red blood in gastric aspirate that did not clear after tube adjustment and 10-min lavage (2) Persistent coffee ground material for 8 h that did not clear with lavage, or accompanied by 5 % decrease in Hct (3) Decrease in Hct requiring ≥1 transfusions in the absence of obvious source or (4) Melena or hematochizia	Radiological changes	Pharmaceutical
Fink [33] Abstract USA (*n* = 189)	Adult critically ill patients Age NR Male NR APACHE II 15	Pantoprazole 40 mg IV daily, 40 mg IV twice daily, 80 mg IV daily, or 80 mg IV twice daily (*n* = 158); Cimetidine IV 300 mg bolus, then 50 mg/h infusion (*n* = 31)	No clear definition	NA	NR
Bashar [21] Iran (*n* = 120)	MV trauma patients, APACHE II < 25 Age 40.15 Male 7 % APACHE II 15.2	Pantoprazole 40 mg IV daily then 40 mg PO daily when enteral feeds started (*n* = 60) Ranitidine 50 mg IV three times daily while NPO then 150 mg PO daily when enteral feeds started (*n* = 60)	No clear definition	Clinical Pulmonary Infection Score (CPIS)	NR
Lee [23] Taiwan (*n* = 60)	Neurosurgical ICU Age 57.7 years Male 60 % APACHE II 17.1	Esomeprazole 40 mg PO daily for 7 days (*n* = 30) Famotidine 20 mg IV twice daily for 7 days (*n* = 30)	Overt bleeding, or decreased hemoglobin level >2 g/dL and lesions on endoscopy	>48 h of ventilation and 3 or more of: (1) Persistent (>48 h) or new infiltrate (2) Positive sputum smear (3) Fever >38.3 °C (4) WBC >12 × 10^9^/L	Academic
Liu [24] China (*n* = 165)	Neurosurgical ICU with ICH Age NA Male 65 (58 %) APACHE II NR	Omeprazole 40 mg IV twice daily (*n* = 58) Cimetidine 300 mg IV four times daily (*n* = 54) Placebo (*n* = 53)	Overt bleeding that requires transfusion, with or without hemodynamic instability	NR	Academic
Fogas [22] Abstract Hungary (*n* = 79)	MV patients Age 69.5 Male 61 % APACHE II 27	PPI (*n* = 38) H2RA (*n* = 41) No molecule, route, dose or frequency described	No clear definition	Leukocytosis, elevated procalcitonin, fever, purulent ETT secretion, positive ETT microbiology, new/increased infiltrate	NR
Wee [20] Abstract USA (n =129)	Critically ill patients with risk factors Age median 72 Male NR APACHE II 22	Pantoprazole 40 mg IV daily (*n* = 68) Famotidine 20 mg IV twice daily (*n* = 61)	Overt bleeding with any of the following: (1) Decrease in SBP by >20 mmHg (2) Decrease in MAP to <65 mmHg 3. Decrease in Hb >2 g/dL and need for >1 unit of blood	NA	NR
Bhanot [38] Abstract India (*n* = 150)	Mechanically ventilated, critically ill.	Omeprazole 40 mg PO daily (*n* = 50) Ranitidine 50 mg IV four times daily (*n* = 50) Sucralfate 1 gm PO four times daily (*n* = 50)	NR	NR	NR

Description of populations, settings, interventions, outcomes and funding sources. *APACHE* Acute Physiology and Chronic Health Evaluation, *MV* mechanically ventilated, *NR* not reported, *GI* gastrointestinal, *IV* intravenous, *PO* oral, *hb* hemoglobin, *USFDA* US Food and Drug Agency, *SBP* systolic blood pressure, *HR* heart rate, *ETT* endotracheal tube, *WBC* white blood cells, *BAL*, bronchiolar lavage, *CFU* colony-forming units, *DBP* diastolic blood pressure, *Hct* hematocrit, *PPI* proton pump inhibitor, *H2RA* histamine-2-receptor antagonist, *MAP* mean arterial pressure, *CDC* Center of disease control, *NG* nasogastric, *NA* not applicable

### Risk of bias assessment

Using the Cochrane risk of bias tool, three trials were judged to be at low risk of bias, and for six trials the risk of bias was unclear (Additional file 1: Table S6). We could not evaluate the risk of bias in six trials published as abstracts [20, 23, 32–34, 38]. In total, 10 trials were judged to be at high risk of bias, primarily due to lack of or inappropriate blinding.

### Assessment of quality of the evidence

We used the GRADE method [26] to assess the quality of evidence for individual outcomes. We present the details of our assessment in Table 2.

**Table 2:** Grading of Recommendation, Assessment, Development, and Evaluation (GRADE) evidence profile

Quality assessment						Patients, number		Effect		Quality
Studies, number	Risk of bias	Inconsistency	Indirectness	Imprecision	Other considerations	PPIs	H2RAs	Relative (95 % CI)	Absolute (95 % CI)	
Clinically important bleeding										
14	Serious^a^	Not serious	Not serious	Not serious^b^	None	13/986 (1.3 %)	39/693 (5.6 %)	RR 0.39 (0.21, 0.71)	15 fewer per 1000 (7–20 fewer)	Moderate^a,b^
Overt upper gastrointestinal bleeding										
17	Serious^a^	Not serious^c^	Not serious	Not serious	None	53/1102 (4.8 %)	118/795 (14.8 %)	RR 0.48 (0.34, 0.66)	26 fewer per 1000 (17–33 fewer)	Moderate^a,c^
Nosocomial pneumonia										
13	Serious^a^	Not serious^d^	Not serious	Serious^e^	None	119/862 (13.8 %)	92/709 (13.0 %)	RR 1.12 (0.86, 1.46)	16 more per 1000 (18 fewer to 60 more)	Low^a,d,e^
Mortality										
11	Not serious^f^	Not serious	Not serious	Serious^e^	None	151/874 (17.3 %)	120/614 (19.5 %)	RR 1.05 (0.87, 1.27)	10 more per 1000 (25 fewer to 53 more)	Moderate^e,f^
ICU length of stay										
7	Serious^g^	Not serious	Not serious	Serious^h^	None	371	373	-	MD 0.58 days fewer (2.03 fewer to 0.86 more)	Low^g,h^

The Guideline Development Tool was used to summarize the quality of evidence for individual outcomes based on five main domains: risk of bias, inconsistency, indirectness, imprecision, and publication bias. *PPI* proton pump inhibitor, *H2RA* histamine-2-receptor antagonist, *MD* mean difference, *RR* relative risk. ^a^We downgraded by one level, for risk of bias; most studies were unblinded. ^b^Although the total number of events was small, we did not downgrade for imprecision. ^c^Significant inconsistency was not present (*I* ^2^ = 6 %). ^d^Significant inconsistency was not present (*I* ^2^ = 4 %). ^e^We downgraded by one level for imprecision; the confidence interval contains significant benefit and harm. ^f^We did not downgrade for risk of bias because mortality is an objective outcome that is less likely to be affected by lack of blinding in clinical trials. ^g^We downgraded by one level for risk of bias. ^h^We downgraded by one level for imprecision; the confidence interval contained significant benefit and harm

### Main outcomes

**Fig. 2:**
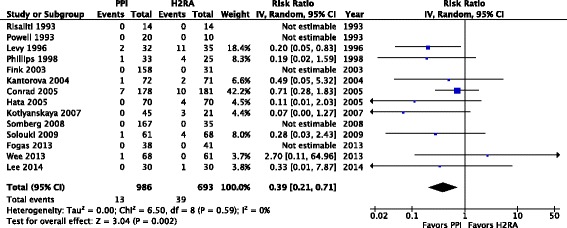
Clinically important gastrointestinal bleeding. Data from 14 trials (*n* = 1679 patients) are included, analyzed using the random effects model. Proton pump inhibitors (*PPIs*) were associated with a significantly lower risk of clinically important bleeding compared to histamine-2-receptor antagonists (*H2RAs*). *IV* Inverse Variance

**Fig. 3:**
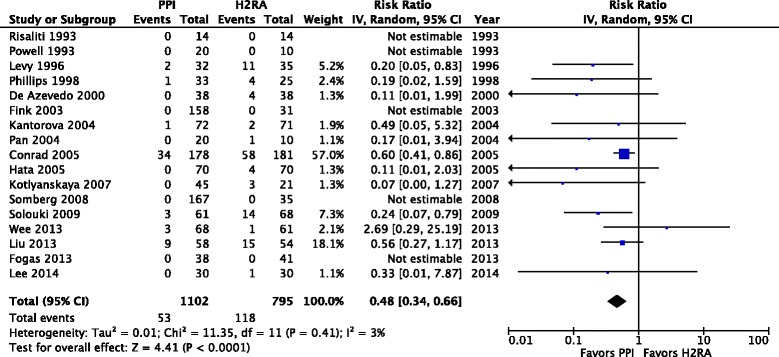
Overt upper gastrointestinal bleeding. Data from 17 trials (*n* = 1897 patients) are included, analyzed using the random effects model. Proton pump inhibitors (*PPIs*) were associated with a significantly lower risk of overt bleeding compared to histamine-2-receptor antagonists (*H2RAs*). *IV* Inverse Variance

**Fig. 4:**
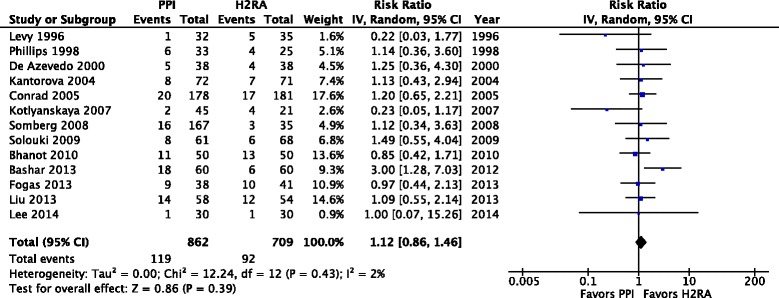
Pneumonia outcome. Data from 12 trials (*n* = 1471 patients) were included, analyzed using the random effects model. The risk of pneumonia was similar in both groups. *PPI* proton pump inhibitor, *H2RA* histamine-2-receptor antagonist. *IV* Inverse Variance

A total of 14 trials enrolling 1679 patients reported clinically important GI bleeding (Fig. 2). PPI use was associated with lower risk of clinically important GI bleeding compared to H2RAs (RR 0.39; 95 % CI 0.21, 0.71; *P* = 0.002; *I*^*2*^ = 0 %; moderate confidence). Using an assumed control event rate of 3 %, the number needed to treat (NNT) was 55 (95 % CI 42, 115). Seventeen trials enrolling 1897 patients reported overt GI bleeding (Fig. 3). Prophylaxis with PPI was associated with a lower risk of overt GI bleeding compared to H2RA (RR 0.48; 95 % CI 0.34, 0.66; *P* < 0.0001; *I*^*2*^ = 3 %, moderate confidence). The NNT to prevent GI bleeding was 37 (95 % CI 29, 59) for an assumed control event rate of 5 %. Thirteen trials enrolling 1571 patients reported the risk of pneumonia (Fig. 4). The risk of pneumonia was similar between groups (RR 1.12; 95 % CI 0.86, 1.46; *P* = 0.39; *I*^*2*^ = 2 %, low confidence). Eleven trials enrolling 1487 patients reported on mortality (Additional file 1: Figure S5). Mortality risk was similar between groups (RR 1.05; 95 % CI 0.87, 1.27; *P* = 0.61; *I*^*2*^ = 0 %, moderate confidence). Seven trials enrolling 744 patients reported ICU length of stay (Additional file 1: Figure S6); ICU length of stay was not significantly different between groups (MD –0.38 days; 95 % CI –1.49, 0.74; *P* = 0.51; *I*^*2*^ = 30 %, low confidence). None of the RCTs included reported on *Clostridium difficile* infection.

### Subgroup analyses

We found no statistically significant interaction between the magnitude of effect and risk of bias, route of PPI administration, or frequency of PPI dosing. Details of the results of the subgroup analyses are in Additional file 1: Table S4 and S5.

Sensitivity analysis excluding trials published as abstracts yielded results very similar to the primary analysis (RR 0.42; 95 % CI 0.21, 0.82; *P* = 0.01; *I*^*2*^ = 0 %) (Additional file 1: Figure S7).

### Publication bias

Visual inspection of the funnel plot for clinically important GI bleeding did not suggest the presence of publication bias (Additional file 1: Figure S8). The Egger test also supported this conclusion (–0.69; 95 % CI –2.44, 0.84; *P* = 0.28). The Egger test was significant for overt GI bleeding, which may suggest the presence of publication bias (–0.87; 95 % CI –1.67, –0.07; *P* = 0.03). We did not find evidence of publication bias for the outcomes of mortality and pneumonia (Additional file 1: Figure S9 and S10).

## Discussion

This systematic review demonstrated moderate quality evidence that PPIs are superior to H2RAs in reducing the risk of both clinically important and overt GI bleeding. The relative treatment effect was large (relative risk reduction of 61 % for clinically important GI bleeding), and the NNT was 55, which translates into 16 fewer bleeding events per 1000 patients. The relatively low incidence of GI bleeding currently seen in the ICU explains the apparent discrepancy between a large relative effect and small absolute effect (Table 2).

The primary concern associated with PPI use in the ICU is the potentially higher risk of infection, particularly, pneumonia and *C. difficile* [19], potentially a consequence of attenuation of the gastric acid protection against bacteria. Patients with achlorhydria or on long-term acid suppressive therapy have increased bacterial growth on gastric mucosal biopsy [49]. Whether this translates into increased risk of infection in critically ill patients remains unknown.

Our meta-analysis did not suggest an increased risk of pneumonia associated with PPIs rather than H2RAs. Furthermore, mortality and duration of stay in the ICU did not differ between groups. The impact of acid suppressive therapy on *Clostridium difficile* infection is uncertain as none of the included trials reported on this. A systematic review of 12 observational studies evaluating 2948 non-ICU patients with *Clostridium difficile* found an association with acid suppressive therapy (OR 1.94; 95 % CI 1.37, 2.75). This association was larger with PPI use (OR 2.05; 95 % CI 1.47, 2.85) as compared to H2RA (OR 1.47; 95 % CI 1.06, 2.05), but the difference was not statistically significant (*P* = 0.17) [50]. Furthermore, a retrospective cohort study that used propensity matching and included over 30,000 critically ill patients suggested that PPI use was associated with a small increase in the risk of *Clostridium difficile* infection in comparison to H2RA (3.4 % vs. 2.7 %; *P* = 0.02) [19]. As these results are limited by risk of bias associated with observational designs and imprecision, randomized trials are needed to confirm or refute these observations.

Our meta-analysis used broad eligibility criteria to enhance the generalizability of the results. Despite including a wide spectrum of critically ill patients, there was no significant heterogeneity across trials for these outcomes. Our findings suggest that PPIs are effective in preventing stress ulcer bleeding without increasing the risk of pneumonia or mortality. Nonetheless, several factors suggest cautious interpretation of these results. The quality of evidence was moderate for most outcomes; this is primarily related to risk of bias. Nine trials did not employ proper blinding of healthcare providers or outcome assessors (Additional file 1: Table S6), which may have inflated the observed treatment effect of PPIs. Furthermore, a subgroup analysis based on risk of bias (low vs. high or unclear) suggested a larger treatment effect in trials of lower quality, although the test for interaction was not significant. The outcome definitions varied across studies, which may have affected the estimate of effect.

Other factors, such as enteral nutrition, may modify the efficacy of prophylactic PPIs and H2RAs. One small RCT in critically ill patients with burns showed that enteral nutrition increased gastric blood flow [51]. No RCTs exclusively examined the effect of enteral feeding on bleeding from stress ulcers. Whether enteral feeding modifies the effect of PPIs on the risk of bleeding or pneumonia remains uncertain.

Prior meta-analyses examined the effect of PPIs compared to H2RAs for stress ulcer prophylaxis [11–14]. Our meta-analysis included more trials than other meta-analyses on this topic (19 trials enrolling 2117 patients), improving the precision of our findings. We obtained additional missing data from the authors of the original trial reports to inform the analyses, and conducted subgroup analyses to assess the robustness of the findings. Using the GRADE approach to assess our confidence in the estimates of treatment effect, the certainty of evidence was moderate for clinically important and overt upper GI bleeding, and mortality outcomes, while it was low for pneumonia and ICU length of stay outcomes. We also adhered to the Preferred Reporting Items for Systematic Reviews and Meta-analyses (PRISMA) reporting guidelines [52].

## Conclusions

In summary, our meta-analysis provides moderate quality evidence for clinicians and guideline groups suggesting that PPIs, when compared to H2RAs, lower the risk of clinically important and overt GI bleeding among critically ill patients, without increasing the risk of pneumonia and mortality, or ICU length of stay. The impact of these drugs on the risk of *Clostridium difficile* infection has yet to be examined in randomized trials in the ICU setting.

## Additional file

Additional file 1: Figure S5. Forest plot for ICU mortality. Data from 11 trials (*n* = 1487 patients) were included, analyzed using the random effects model. The risk of death during the ICU stay was similar in both groups. **Figure S6.** Forest plot for ICU length of stay. Data from 11 trials (*n* = 744 patients) were included, analyzed using the random effects model. The duration of ICU stay was similar in both groups. **Figure S7.** Sensitivity analysis for clinically important bleeding, excluding trials published as abstracts, shows similar results to the primary analysis. **Figure S8.** Funnel plot for clinically important bleeding outcome. Visual inspection does not show publication bias and the result for the Egger test was –0.69 (95 % CI –2.44, 0.84; *P* = 0.28). **Figure S9.** Funnel plot for pneumonia outcome. Visual inspection does not show publication bias. **Figure S10.** Funnel plot for ICU mortality outcome. Visual inspection does not show publication bias. (DOCX 1123 kb)
