# Supplementary material for: Efficacy and safety of proton pump inhibitors for stress ulcer prophylaxis in critically ill patients: a systematic review and meta-analysis of randomized trials
Source: Crit Care. 2016 May 4;20:120. doi: 10.1186/s13054-016-1305-6 (PMC4855320; doi:10.1186/s13054-016-1305-6)
Supplement: Additional file 1: Figure S5. — Forest plot for ICU mortality. Data from 11 trials (n = 1487 patients) were included, analyzed using the random effects model. The risk of death during the ICU stay was similar in both groups. Figure S6. Forest plot for ICU length of stay. Data from 11 trials (n = 744 patients) were included, analyzed using the random effects model. The duration of ICU stay was similar in both groups. Figure S7. Sensitivity analysis for clinically important bleeding, excluding trials published as abstracts, shows similar results to the primary analysis. Figure S8. Funnel plot for clinically important bleeding outcome. Visual inspection does not show publication bias and the result for the Egger test was –0.69 (95 % CI –2.44, 0.84; P = 0.28). Figure S9. Funnel plot for pneumonia outcome. Visual inspection does not show publication bias. Figure S10. Funnel plot for ICU mortality outcome. Visual inspection does not show publication bias. (DOCX 1123 kb) [file 13054_2016_1305_MOESM1_ESM.docx]

**Efficacy and Safety of Proton Pump Inhibitors for Stress Ulcer Prophylaxis in Critically ill patients: A Systematic Review and Meta-analysis of Randomized Trials**

**Authors**

Fayez Alshamsi, Emilie Belley-Cote, Deborah Cook, Saleh A. Almenawer, Zuhoor Alqahtani, Dan Perri, Lehana Thabane, Awad Al-Omari, Kim Lewis, Gordon Guyatt, Waleed Alhazzani

e-Appendix

|  |  | Page |
| --- | --- | --- |
|  | e-Table 3: Search Strategy EMBASE and MEDLINE | 2-3 |
|  | e-Table 4: Search Strategy for Cochrane Central Register of Controlled Trials | 4-6 |
|  | e-Table 5: Search Strategy for ACP Journal Club | 6-7 |
|  | e-Table 6: Risk of Bias assessment of the included trials using Cochrane Risk of Bias tool. | 8 |
|  | e-Figure 5: Forest Plot for ICU Mortality. | 9 |
|  | e-Figure 6: Forest Plot for ICU Length of Stay. | 9 |
|  | e-Table 7: Subgroup Analysis for Clinically Important Bleeding outcome. | 10 |
|  | e-Table 8: Subgroup Analysis for Overt Upper Gastrointestinal Bleeding outcome. | 10 |
|  | e-Figure 7: Sensitivity analysis for Clinically Important Bleeding. | 11 |
|  | e-Figure 8: Funnel Plot for Clinically Important Bleeding Outcome | 12 |
|  | e-Figure 9: Funnel plot for pneumonia outcome. | 13 |
|  | e-Figure 10: Funnel plot for ICU mortality outcome. | 14 |

**e-Table 3**: Search Strategy EMBASE and MEDLINE

| **#** | **Searches** | **Results** |
| --- | --- | --- |
| 1 | Proton Pump Inhibitors/ or (proton adj5 pump adj5 inhibitor*).mp. or ppi*.tw. or exp Omeprazole/ or Benzimidazoles/ or Omeprazole.mp. or losec.mp. or omez.mp. or prilosec.mp. or rapinex.mp. or zegerid.mp. or esomeprazole.mp. or axagon.mp. or esporal.mp. or lucen.mp. or nexiam.mp. or nexium.mp. or sompraz.mp. or soleri.mp. or Lansoprazole.mp. or agopton.mp. or bamalite.mp. or lansoprazole.mp. or lanzor.mp. or monolitum.mp. or ogast.mp. or ogastro.mp. or ogastro.mp. or opiren.mp. or prevacid.mp. or prezal.mp. or pro ulco.mp. or promeco.mp. or takepron.mp. or ulpax.mp. or zoton.mp. or 2-Pyridinylmethylsulfinylbenzimidazoles/ or dexlansoprazole.mp. or kapidex.mp. or dexilant.mp. or pantoprazole.mp. or pantoloc.mp. or pantecta.mp. or protium.mp. or protonix.mp. or rabeprazole.mp. or AcipHex.mp. or Pariet.mp. or dexrabeprazole.mp. or rablet.mp. [mp=ti, ab, hw, tn, ot, dm, mf, dv, kw, nm, kf, px, rx, ui] | 132847 |
| 2 | exp Histamine H2 Antagonists/ or ((H2 or histamine) adj3 (blocker* or blockader*)).mp. or ((H2 or histamine) adj3 (receptor* or antagonist*)).mp. or (H2 adj1 antihistaminic*).mp. or H2RA*.mp. or exp Cimetidine/ or Guanidines/ or Imidazoles/ or cimetidine.mp. or acitak.mp. or altramet.mp. or biomet.mp. or dyspamet.mp. or eureceptor.mp. or galenamet.mp. or histodil.mp. or peptimax.mp. or phimetin.mp. or tagamet.mp. or ultec.mp. or zita.mp. or FAMOTIDINE/ or famotidine.mp. or fluxid.mp. or leader acid reducer.mp. or mylanta.mp. or pepcid.mp. or Thiazoles/ or roxatidine.mp. or Roxit.mp. or NIZATIDINE/ or Nizatidine.mp. or axid.mp. or tazac.mp. or zinga.mp. or RANITIDINE/ or ranitidine.mp. or azanplus.mp. or biotidin.mp. or pylorid.mp. or raciran.mp. or raniberl.mp. or ranisen.mp. or ranitidin.mp. or rantec.mp. or sostril.mp. or taladine.mp. or tritec.mp. or wal-zan.mp. or zantac.mp. or Furans/ [mp=ti, ab, hw, tn, ot, dm, mf, dv, kw, nm, kf, px, rx, ui] | 273683 |
| 3 | exp Peptic Ulcer/ or Ulcer*.mp. or lesion*.mp. | 2096921 |
| 4 | (bleed* or re-bleed* or rebleed* or h?emorrhag*).mp. | 1061059 |
| 5 | 3 and 4 | 143622 |
| 6 | (stress adj3 ulcer*).mp. | 7469 |
| 7 | 5 or 6 | 148693 |
| 8 | exp Gastrointestinal Hemorrhage/ | 126523 |
| 9 | ((gastrointestinal or gastro-intestinal) adj5 (bleed* or re-bleed* or rebleed* or h?emorrhag*)).mp. | 112306 |
| 10 | (h?ematochezia* or h?ematemese* or mel?ena*).mp. | 17603 |
| 11 | exp injury/ and exp gastric mucosa/ | 3344 |
| 12 | (mucos* adj5 injur*).tw. | 13118 |
| 13 | (stomach or antrum or antral or pyloric or pylorus or gastri* or gastropathy or epigastr* or duodenal or duodenum or gastro-duodenal or gastroduodenal or oeso*ag* or esp*ag* or "upper GI" or UGI or "upper gastrointestinal" or "upper gastrointestinal").mp. | 1113960 |
| 14 | 4 and 13 | 99680 |
| 15 | exp Gastritis/ | 76781 |
| 16 | 4 and 15 | 6223 |
| 17 | or/7-12,14,16 | 289683 |
| 18 | exp Critical Care/ | 559710 |
| 19 | exp intensive care/ | 534305 |
| 20 | exp Critical Illness/ | 42894 |
| 21 | exp Intensive Care Units/ | 165170 |
| 22 | ICU*.tw. | 107408 |
| 23 | ((critical or intensive) adj3 (care or illness)).tw. | 294703 |
| 24 | exp Intubation, Gastrointestinal/ | 14809 |
| 25 | exp Monitoring, Physiologic/ | 144136 |
| 26 | exp Multiple Organ Failure/ | 36501 |
| 27 | exp Acid-Base Equilibrium/ | 34737 |
| 28 | exp Multiple Trauma/ | 22099 |
| 29 | (serious* adj injur*).tw. | 8026 |
| 30 | (severe adj (traum* or shock)).tw. | 15392 |
| 31 | exp Perioperative Care/ | 163356 |
| 32 | ((preoperative or intraoperative or perioperative) adj (care or procedure* or period)).tw. | 24896 |
| 33 | exp Resuscitation/ | 162860 |
| 34 | exp Shock/ | 167096 |
| 35 | exp sepsis/ | 292378 |
| 36 | exp Ventilator Weaning/ | 144800 |
| 37 | exp Ventilators, Mechanical/ | 9597 |
| 38 | exp Ventilators, Negative-Pressure/ | 20024 |
| 39 | (protocol* adj weaning).mp. [mp=ti, ab, hw, tn, ot, dm, mf, dv, kw, nm, kf, px, rx, ui] | 97 |
| 40 | (ventilat* adj weaning).mp. [mpP=title, original title, abstract, name of substance word, subject heading word] | 4199 |
| 41 | ((artificial or mechanical) adj ventilat*).mp. | 140789 |
| 42 | ventilat*.tw. | 312929 |
| 43 | or/18-42 | 1739921 |
| 44 | randomized controlled trial.pt. or Randomi?ed Controlled trial*.mp. or Randomi?ed clinical trial*.mp. or Random Allocation/ or Random allocation.mp. or random*.tw. or clinical trial/ or controlled clinical trial/ or single-blind method/ or double-blind method/ or ((singl* or doubl* or trip* or trebl*) adj25 (blind* or mask*)).mp. or Placebos/ or placebo*.tw. or drug therapy.fs. or trial.ab. or groups.ab. | 7608212 |
| 45 | 1 or 2 | 384217 |
| 46 | 17 and 43 and 44 and 45 | 1130 |
| 47 | limit 46 to ed=20150401-20151125 [Limit not valid in Embase; records were retained] | 779 |
| 48 | limit 47 to dd=20150401-20151125 [Limit not valid in Ovid MEDLINE(R),Ovid MEDLINE(R) Daily Update,Ovid MEDLINE(R) In-Process; records were retained] | 35 |
| 49 | remove duplicates from 48 | 30 |

**e-Table 4:** Search Strategy for Cochrane Central Register of Controlled Trials

| **#** | **Searches** | **Results** |
| --- | --- | --- |
| 1 | Proton Pump Inhibitors/ or (proton adj5 pump adj5 inhibitor*).mp. or ppi*.tw. or exp Omeprazole/ or Benzimidazoles/ or Omeprazole.mp. or losec.mp. or omez.mp. or prilosec.mp. or rapinex.mp. or zegerid.mp. or esomeprazole.mp. or axagon.mp. or esporal.mp. or lucen.mp. or nexiam.mp. or nexium.mp. or sompraz.mp. or soleri.mp. or Lansoprazole.mp. or agopton.mp. or bamalite.mp. or lansoprazole.mp. or lanzor.mp. or monolitum.mp. or ogast.mp. or ogastro.mp. or ogastro.mp. or opiren.mp. or prevacid.mp. or prezal.mp. or pro ulco.mp. or promeco.mp. or takepron.mp. or ulpax.mp. or zoton.mp. or 2-Pyridinylmethylsulfinylbenzimidazoles/ or dexlansoprazole.mp. or kapidex.mp. or dexilant.mp. or pantoprazole.mp. or pantoloc.mp. or pantecta.mp. or protium.mp. or protonix.mp. or rabeprazole.mp. or AcipHex.mp. or Pariet.mp. or dexrabeprazole.mp. or rablet.mp. [mp=title, abstract, subject headings, heading word, drug trade name, original title, device manufacturer, drug manufacturer, device trade name, keyword] | 6350 |
| 2 | exp Histamine H2 Antagonists/ or ((H2 or histamine) adj3 (blocker* or blockader*)).mp. or ((H2 or histamine) adj3 (receptor* or antagonist*)).mp. or (H2 adj1 antihistaminic*).mp. or H2RA*.mp. or exp Cimetidine/ or Guanidines/ or Imidazoles/ or cimetidine.mp. or acitak.mp. or altramet.mp. or biomet.mp. or dyspamet.mp. or eureceptor.mp. or galenamet.mp. or histodil.mp. or peptimax.mp. or phimetin.mp. or tagamet.mp. or ultec.mp. or zita.mp. or FAMOTIDINE/ or famotidine.mp. or fluxid.mp. or leader acid reducer.mp. or mylanta.mp. or pepcid.mp. or Thiazoles/ or roxatidine.mp. or Roxit.mp. or NIZATIDINE/ or Nizatidine.mp. or axid.mp. or tazac.mp. or zinga.mp. or RANITIDINE/ or ranitidine.mp. or azanplus.mp. or biotidin.mp. or pylorid.mp. or raciran.mp. or raniberl.mp. or ranisen.mp. or ranitidin.mp. or rantec.mp. or sostril.mp. or taladine.mp. or tritec.mp. or wal-zan.mp. or zantac.mp. or Furans/ [mp=title, abstract, subject headings, heading word, drug trade name, original title, device manufacturer, drug manufacturer, device trade name, keyword] | 10886 |
| 3 | exp Peptic Ulcer/ or Ulcer*.mp. or lesion*.mp. | 29937 |
| 4 | (bleed* or re-bleed* or rebleed* or h?emorrhag*).mp. | 24655 |
| 5 | 3 and 4 | 2251 |
| 6 | (stress adj3 ulcer*).mp. | 222 |
| 7 | 5 or 6 | 2348 |
| 8 | exp Gastrointestinal Hemorrhage/ | 1532 |
| 9 | ((gastrointestinal or gastro-intestinal) adj5 (bleed* or re-bleed* or rebleed* or h?emorrhag*)).mp. | 2326 |
| 10 | (h?ematochezia* or h?ematemese* or mel?ena*).mp. | 166 |
| 11 | exp injury/ and exp gastric mucosa/ | 10 |
| 12 | (mucos* adj5 injur*).tw. | 327 |
| 13 | (stomach or antrum or antral or pyloric or pylorus or gastri* or gastropathy or epigastr* or duodenal or duodenum or gastro-duodenal or gastroduodenal or oeso*ag* or esp*ag* or "upper GI" or UGI or "upper gastrointestinal" or "upper gastrointestinal").mp. | 23638 |
| 14 | 4 and 13 | 2490 |
| 15 | exp Gastritis/ | 518 |
| 16 | 4 and 15 | 42 |
| 17 | or/7-12,14,16 | 5011 |
| 18 | exp Critical Care/ | 1498 |
| 19 | exp intensive care/ | 938 |
| 20 | exp Critical Illness/ | 928 |
| 21 | exp Intensive Care Units/ | 2117 |
| 22 | ICU*.tw. | 3275 |
| 23 | ((critical or intensive) adj3 (care or illness)).tw. | 8057 |
| 24 | exp Intubation, Gastrointestinal/ | 528 |
| 25 | exp Monitoring, Physiologic/ | 8498 |
| 26 | exp Multiple Organ Failure/ | 296 |
| 27 | exp Acid-Base Equilibrium/ | 380 |
| 28 | exp Multiple Trauma/ | 165 |
| 29 | (serious* adj injur*).tw. | 66 |
| 30 | (severe adj (traum* or shock)).tw. | 375 |
| 31 | exp Perioperative Care/ | 9451 |
| 32 | ((preoperative or intraoperative or perioperative) adj (care or procedure* or period)).tw. | 1059 |
| 33 | exp Resuscitation/ | 3249 |
| 34 | exp Shock/ | 1198 |
| 35 | exp sepsis/ | 2630 |
| 36 | exp Ventilator Weaning/ | 351 |
| 37 | exp Ventilators, Mechanical/ | 208 |
| 38 | exp Ventilators, Negative-Pressure/ | 25 |
| 39 | (protocol* adj weaning).mp. [mp=title, abstract, subject headings, heading word, drug trade name, original title, device manufacturer, drug manufacturer, device trade name, keyword] | 12 |
| 40 | (ventilat* adj weaning).mp. [mpP=title, original title, abstract, name of substance word, subject heading word] | 424 |
| 41 | ((artificial or mechanical) adj ventilat*).mp. | 3730 |
| 42 | ventilat*.tw. | 12769 |
| 43 | or/18-42 | 41915 |
| 44 | randomized controlled trial.pt. or Randomi?ed Controlled trial*.mp. or Randomi?ed clinical trial*.mp. or Random Allocation/ or Random allocation.mp. or random*.tw. or clinical trial/ or controlled clinical trial/ or single-blind method/ or double-blind method/ or ((singl* or doubl* or trip* or trebl*) adj25 (blind* or mask*)).mp. or Placebos/ or placebo*.tw. or drug therapy.fs. or trial.ab. or groups.ab. | 590360 |
| 45 | 1 or 2 | 16054 |
| 46 | 17 and 43 and 44 and 45 | 143 |
| 47 | limit 46 to yr="2011 -Current" | 15 |
| 48 | limit 46 to yr="2014 -Current" | 3 |

**e-Table 5:** Search Strategy for ACP Journal Club

| **#** | **Searches** | **Results** |
| --- | --- | --- |
| 1 | (proton adj5 pump adj5 inhibitor*).mp. | 75 |
| 2 | ppi*.tw. | 40 |
| 3 | (Omeprazole or losec or omez or prilosec or rapinex or zegerid).mp. | 65 |
| 4 | (esomeprazole or axagon or esporal or lucen or nexiam or nexium or sompraz or soleri).mp. | 12 |
| 5 | (Lansoprazole or agopton or bamalite or lansoprazole or lanzor or monolitum or ogast or ogastro or ogastro or opiren or prevacid or prezal or pro ulco or promeco or takepron or ulpax or zoton).mp. | 14 |
| 6 | (dexlansoprazole or kapidex or dexilant).mp. | 0 |
| 7 | (pantoprazole or pantoloc or pantecta or protium or protonix).mp. | 6 |
| 8 | (rabeprazole or AcipHex or Pariet or dexrabeprazole or rablet).mp. | 5 |
| 9 | or/1-8 | 108 |
| 10 | ((H2 or histamine) adj3 (blocker* or blockader*)).mp. | 14 |
| 11 | ((H2 or histamine) adj3 (receptor* or antagonist*)).mp. | 36 |
| 12 | (H2 adj1 antihistaminic*).mp. | 1 |
| 13 | H2RA*.mp. | 5 |
| 14 | (cimetidine or acitak or altramet or biomet or dyspamet or eureceptor or galenamet or histodil or peptimax or phimetin or tagamet or ultec or zita).mp. | 10 |
| 15 | (famotidine or fluxid or leader acid reducer or mylanta or pepcid).mp. | 6 |
| 16 | (roxatidine or Roxit).mp. | 0 |
| 17 | (Nizatidine or axid or tazac or zinga).mp. | 2 |
| 18 | (ranitidine or azanplus or biotidin or pylorid or raciran or raniberl or ranisen or ranitidin or rantec or sostril or taladine or tritec or wal-zan or zantac).mp. | 25 |
| 19 | or/10-18 | 58 |
| 20 | Ulcer*.mp. | 270 |
| 21 | (esophagit* adj5 (peptic or reflux)).mp. | 26 |
| 22 | lesion*.mp. | 226 |
| 23 | or/20-22 | 449 |
| 24 | (bleed* or re-bleed* or rebleed* or h?emorrhag*).mp. | 831 |
| 25 | 23 and 24 | 159 |
| 26 | (stress adj3 ulcer*).mp. | 14 |
| 27 | 25 or 26 | 162 |
| 28 | ((gastrointestinal or gastro-intestinal) adj5 (bleed* or re-bleed* or rebleed* or h?emorrhag*)).mp. | 153 |
| 29 | (h?ematochezia* or h?ematemese* or mel?ena*).mp. | 22 |
| 30 | (mucos* adj5 injur*).tw. | 3 |
| 31 | (stomach or antrum or antral or pyloric or pylorus or gastri* or gastropathy or epigastr* or duodenal or duodenum or gastro-duodenal or gastroduodenal or oeso*ag* or esp*ag* or "upper GI" or UGI or "upper gastrointestinal" or "upper gastrointestinal").mp. | 234 |
| 32 | 24 and 31 | 116 |
| 33 | Gastritis.mp. [mp=title, abstract, full text, keywords, caption text] | 22 |
| 34 | 24 and 33 | 8 |
| 35 | or/27-30,32,34 | 269 |
| 36 | ICU*.mp. | 111 |
| 37 | ((critical or intensive) adj3 (care or illness)).mp. | 290 |
| 38 | Nutritional Support.mp. | 10 |
| 39 | ((artificial or force or tube) adj3 (feeding* or nutrition)).mp. | 11 |
| 40 | ((enternal or parenteral) adj3 (feeding* or nutrition)).mp. | 18 |
| 41 | ((Nasogastric or nasointestinal or orogastric or transabdominal) adj2 (intubat* or tube*)).mp. | 13 |
| 42 | ((Gastrointestinal or gastro-intestinal) adj2 Intubation).mp. | 1 |
| 43 | ((patient or physiologic) adj2 monitoring).mp. | 31 |
| 44 | Multiple Organ Failure*.mp. | 4 |
| 45 | (multiple adj3 (wound* or trauma* or injur*)).mp. | 6 |
| 46 | (serious* adj injur*).mp. | 4 |
| 47 | (severe adj (traum* or shock)).mp. | 5 |
| 48 | ((perioperative or intraoperative or postoperative) adj3 (care or procedure* or period)).mp. | 32 |
| 49 | Resuscitation*.mp. | 86 |
| 50 | Shock.mp. | 170 |
| 51 | (circulatory adj3 (failure or collapse)).mp. | 16 |
| 52 | sepsis.mp. [mp=title, abstract, full text, keywords, caption text] | 96 |
| 53 | ventilat*.mp. | 164 |
| 54 | or/36-53 | 620 |
| 55 | 9 or 19 | 129 |
| 56 | 35 and 54 and 55 | 11 |

e-Table 6: Risk of Bias assessment of the included trials using Cochrane Risk of Bias tool.

| Author | Sequence Generation | Allocation Concealment | Blinding | Incomplete Outcome Data | Selective Reporting Bias | Free of Other Bias | Overall Risk of Bias |
| --- | --- | --- | --- | --- | --- | --- | --- |
| Conrad  et al | Low | Low | Low | Low | Low | Low | Low |
| Azevedo  et al | Low | Low | High | Low | Low | Low | High |
| Hata et al | Low | High | High | Low | Low | Low | High |
| Kantorova  et al | Low | Low | Low | Low | Low | Low | Low |
| Kotlyanskaya  et al | Unclear | Unclear | Unclear | Unclear | Unclear | Unclear | Unclear |
| Levy et al | Low | Low | High | Low | Low | Low | High |
| Pan et al | Unclear | Unclear | High | Low | Unclear | Low | High |
| Phillips et al | Unclear | Unclear | Unclear | Unclear | Unclear | Unclear | Unclear |
| Powell  et al | Low | Unclear | Low | Low | Low | Low | Unclear |
| Risaliti et al | Low | Unclear | Unclear | Low | Unclear | Low | Unclear |
| Solouki  et al | Low | Low | Low | Low | Low | Low | Low |
| Somberg  et al | Low | Low | High | Low | Low | Low | High |
| Fink et al | Unclear | Unclear | High | Unclear | Unclear | Unclear | High |
| Bashar et al | Low | Low | Unclear | High | High | Unclear | High |
| Lee et al | Low | Low | High | Low | Unclear | Unclear | High |
| Liu et al | Low | Low | High | High | Low | Unclear | High |
| Fogas et al | Unclear | Unclear | Unclear | Unclear | Unclear | Unclear | Unclear |
| Wee et al | High | High | High | High | Unclear | Unclear | High |
| Bhanot et al | Unclear | Unclear | Unclear | Unclear | Unclear | Unclear | Unclear |


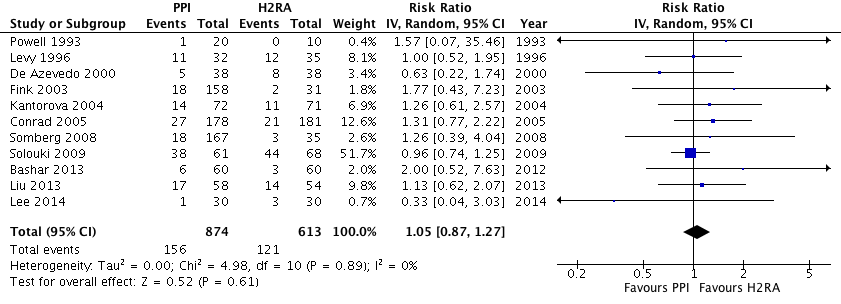


**e-Figure 5:** Forest Plot for ICU Mortality. Data from 11 trials (n=1487) were included, analyzed using the random effects model. The risk of death during the ICU stay was similar in both groups.


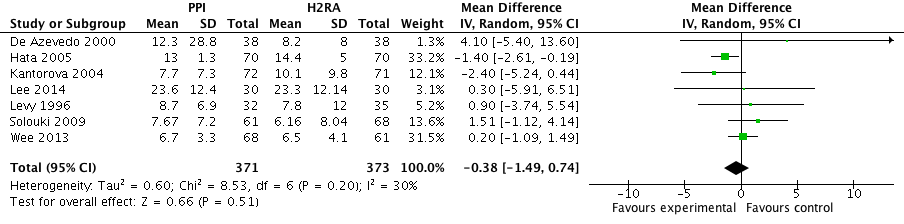


**e-Figure 6:** Forest Plot for ICU Length of Stay. Data from 11 trials (n=744) were included, analyzed using the random effects model. The duration of ICU stay was similar in both groups.

e-Table 7: Subgroup Analysis for Clinically Important Bleeding Outcome

| **Subgroup** | **Subtotal, n** | **Relative Risk**  **(95% Confidence Interval)** | ***P* (Interaction Between Groups)** | ***I^2^* (Heterogeneity Between Groups)** |
| --- | --- | --- | --- | --- |
| Risk of Bias  Low  High/unclear | 631  1048 | 0.60 (0.27, 1.35)  0.22 (0.09, 0.55) | 0.11 | 60.8% |
| Route  Enteral  Parenteral | 907  694 | 0.35 (0.18, 0.67)  0.91 (0.13, 6.09) | 0.35 | 0% |
| Frequency/day  Once  >Once | 984  551 | 0.43 (0.23, 0.84)  0.28 (0.03, 2.43) | 0.70 | 0% |

Measure of treatment effect provided for each subgroup and interaction *P* value and *I^2^* for subgroup difference. All analyses used the inverse variance and fixed effect model.

e-Table 8: Subgroup Analysis for Overt Upper Gastrointestinal Bleeding outcome

| **Subgroup** | **Subtotal, n** | **Relative Risk**  **(95% Confidence Interval)** | ***P* (Interaction Between Groups)** | ***I^2^* (Heterogeneity Between Groups)** |
| --- | --- | --- | --- | --- |
| Risk of Bias  Low  High/unclear | 631  1266 | 0.60 (0.27, 1.35)  0.36 (0.21, 0.63) | 0.32 | 0% |
| Route  Enteral  Parenteral | 937  882 | 0.34 (0.18, 0.64)  0.58 (0.30, 1.12) | 0.25 | 25.0% |
| Frequency/day  Once  >Once | 1014  550 | 0.45 (0.24, 0.84)  0.48 (0.24, 0.94) | 0.90 | 0% |

Measure of treatment effect provided for each subgroup and interaction *P* value and *I^2^* for subgroup difference. All analyses used the inverse variance and fixed effect model.


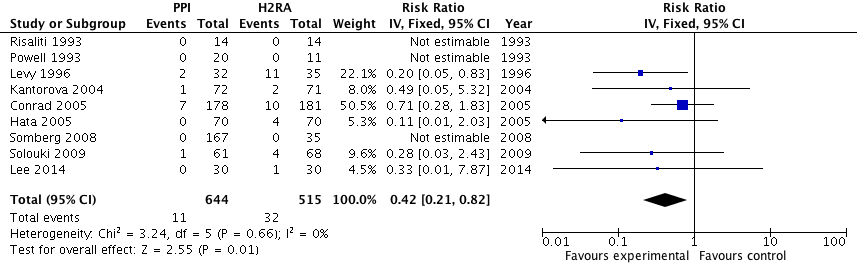


e-Figure 7: Sensitivity analysis for Clinically Important Bleeding excluding trials published as abstracts shows similar results to primary analysis.


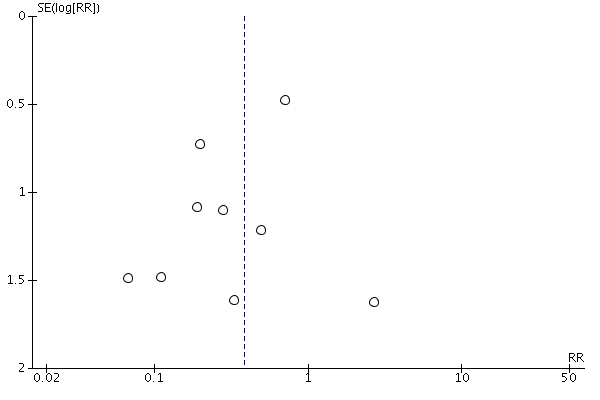


**e-Figure 8:** Funnel Plot for Clinically Important Bleeding Outcome. Visual inspection does not show publication bias and Egger test -0.69 (95% CI -2.44, 0.84; *P*=0.28).


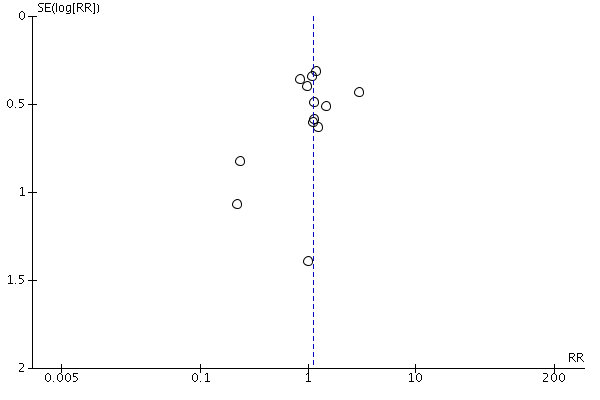


e-Figure 9: Funnel plot for pneumonia outcome. Visual inspection does not show publication bias.


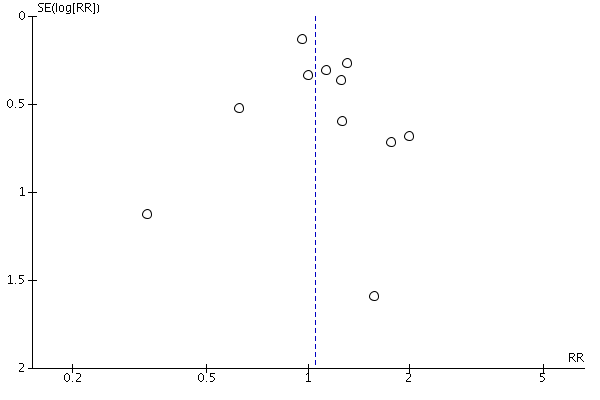


e-Figure 10: Funnel plot for ICU mortality outcome. Visual inspection does not show publication bias.
